# Isolated Pupil-Sparing Third Nerve Palsy Revealing Cryptococcal Meningitis in a Patient With Diabetes Mellitus: A Diagnostic Pitfall

**DOI:** 10.7759/cureus.107674

**Published:** 2026-04-24

**Authors:** Jeeva Shaji

**Affiliations:** 1 Neurology, Believers Church Medical College Hospital, Thiruvalla, IND

**Keywords:** atypical presentation, cranial neuropathy, cryptococcal meningitis, csf cryptococcal antigen, diabetes mellitus, diagnostic pitfall, neuro-ophthalmology, oculomotor nerve palsy, pupil-sparing, third nerve palsy

## Abstract

Isolated pupil-sparing oculomotor nerve palsy is classically attributed to microvascular ischemia, particularly in patients with diabetes mellitus. This heuristic may lead to diagnostic anchoring and delay the identification of serious underlying pathology.

We report a case of an elderly diabetic patient who presented with isolated pupil-sparing oculomotor nerve palsy, a form of cranial neuropathy, and was subsequently found to have cryptococcal meningitis confirmed by positive cerebrospinal fluid (CSF) cryptococcal antigen.

This case highlights that pupil sparing is not a reliable discriminator between ischemic and non-ischemic etiologies. Infectious causes such as cryptococcal meningitis can present atypically as isolated cranial neuropathy, even in the absence of classical meningeal features. Early CSF evaluation is essential in atypical neuro-ophthalmic presentations to avoid diagnostic delay.

## Introduction

Third cranial nerve palsy is a common neuro-ophthalmological condition with a broad differential diagnosis, including microvascular ischemia, compressive lesions, inflammatory disorders, and infections. The presence or absence of pupillary involvement in third nerve palsy remains a critical clinical discriminator for differentiating compressive from microvascular etiologies [1]. In clinical practice, pupil sparing has traditionally been used to favor a diagnosis of ischemic neuropathy, particularly in patients with diabetes mellitus or hypertension, whereas pupil-involving palsy raises concern for compressive lesions such as aneurysms.

Pupil-sparing third nerve palsy is traditionally attributed to microvascular ischemia due to the anatomical arrangement of oculomotor nerve fibers [2]. However, this principle is not absolute and may lead to diagnostic anchoring.

A key learning point is that pupillary involvement is not a binary discriminator but exists along a spectrum influenced by the underlying pathological process. Given the anatomical arrangement of the oculomotor nerve, selective involvement of central somatic fibres with relative sparing of the superficial parasympathetic fibres may occur, potentially mimicking an ischemic pattern [1,3].

Cryptococcal meningitis typically presents with headache, fever, and altered sensorium, especially in immunocompromised individuals, however atypical presentations including isolated cranial nerve palsies are increasingly recognised even in patients even in patients without overt immunosuppression [2,4,5].

Cranial nerve dysfunction in cryptococcal meningitis is thought to arise from a combination of basal meningeal, increased intracranial pressure,and direct fungal invasion [2,4].* *This case illustrates a rare presentation of cryptococcal meningitis as isolated pupil-sparing oculomotor nerve palsy and challenges the conventional diagnostic paradigm.

Another important observation is the relatively benign CSF profile, with minimal pleocytosis and normal biochemical parameters. This underscores that early cryptococcal infection may not exhibit classical inflammatory CSF findings, and reliance on routine parameters alone may be misleading. The detection of cryptococcal antigen remains the cornerstone of diagnosis in such scenarios.

From a clinical practice standpoint, this case reinforces a critical paradigm shift: pupil sparing should not be used in isolation to exclude serious pathology, and clinical reasoning must integrate the entire clinical context rather than rely on traditional heuristics.

The differential diagnosis included diabetic microvascular ischemic neuropathy, posterior communicating artery aneurysm, cavernous sinus pathology, and tuberculous meningitis.

## Case presentation

A 68-year-old South Asian woman with long-standing type 2 diabetes mellitus and hypertension presented with left hemicranial headache, nausea, acute-onset diplopia, and left-sided ptosis of three days' duration. There was no history of fever, altered sensorium, limb weakness, facial numbness, visual blurring, or trauma.

Her diabetes was poorly controlled, with irregular follow-up. There was no history of HIV infection, steroid use, or immunosuppression. Although not classically immunocompromised, poorly controlled diabetes is associated with impaired cell-mediated immunity and increased susceptibility to opportunistic infections.

Neurological examination revealed complete left oculomotor nerve palsy characterized by ptosis, impaired adduction, elevation, and depression of the left eye. The pupil was equal and reactive to light. Fundoscopic examination was normal. The remainder of the cranial nerve and neurological examination was unremarkable.

Investigations

**Figure 1:**
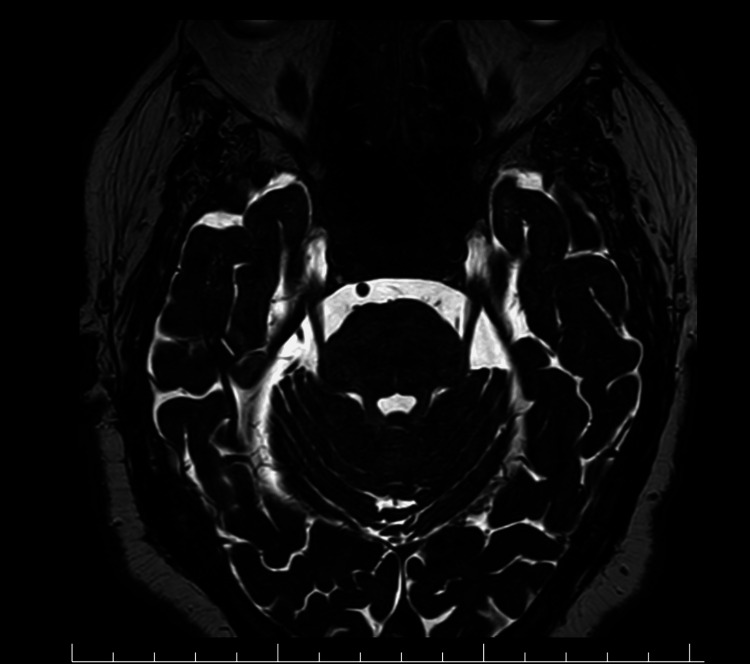
MRI Brain With Contrast Showing No Structural Cause of Third Nerve Palsy Magnetic resonance imaging (MRI) of the brain with contrast demonstrating no evidence of aneurysm, mass lesion, midbrain infarction, or cavernous sinus pathology. No compressive etiology for the left oculomotor nerve palsy is identified.

**Figure 2:**
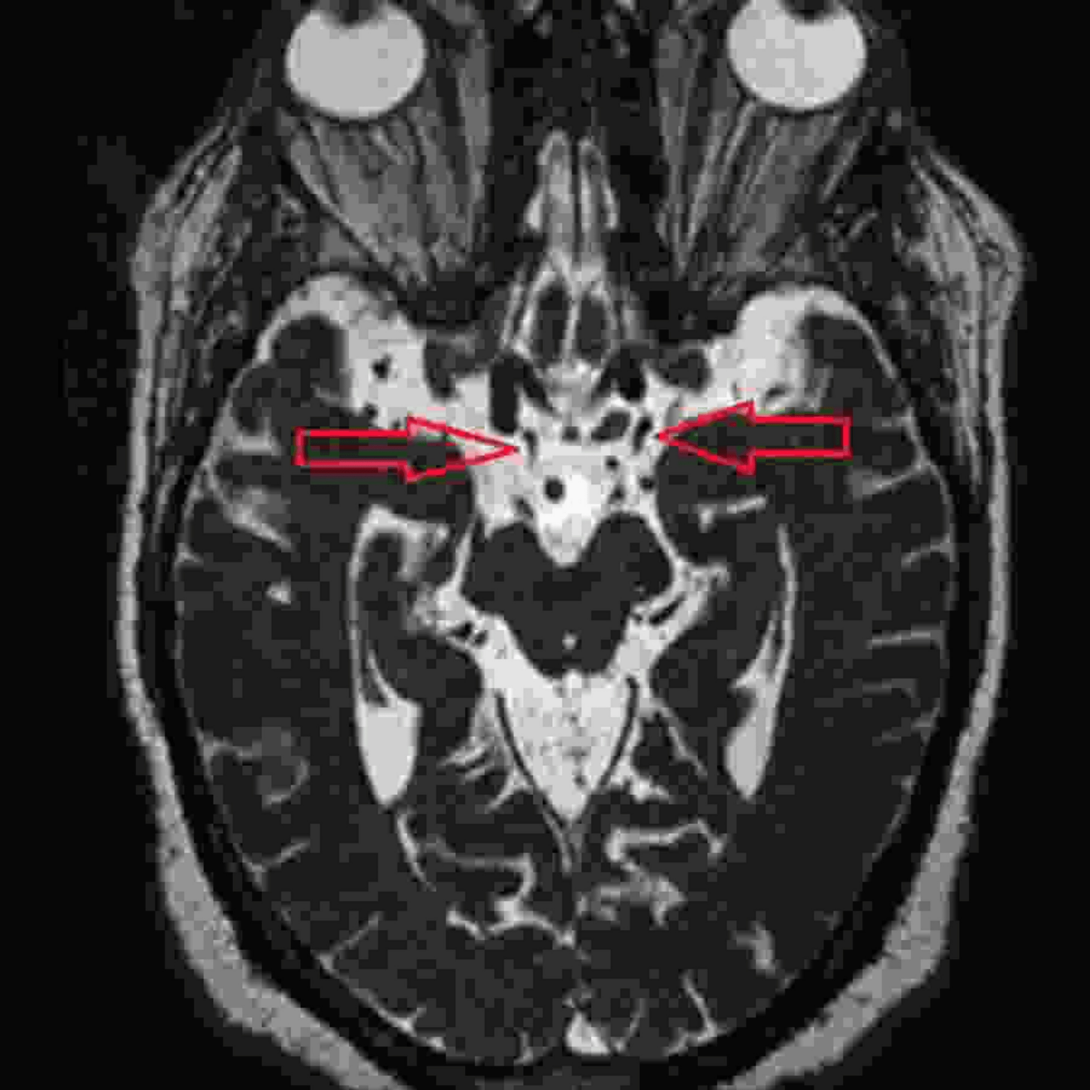
MR Angiography Showing No Posterior Communicating Artery Aneurysm Magnetic resonance angiography (MRA) demonstrating normal intracranial vasculature with no evidence of posterior communicating artery aneurysm or other vascular abnormalities.The arrows show the normal posterior communicating arteries on either side.

Laboratory investigations revealed poor glycemic control. MRI brain with contrast showed no aneurysm, infarction, or cavernous sinus pathology (Figure 1). MR angiography was normal (Figure 2).

**Figure 3:**
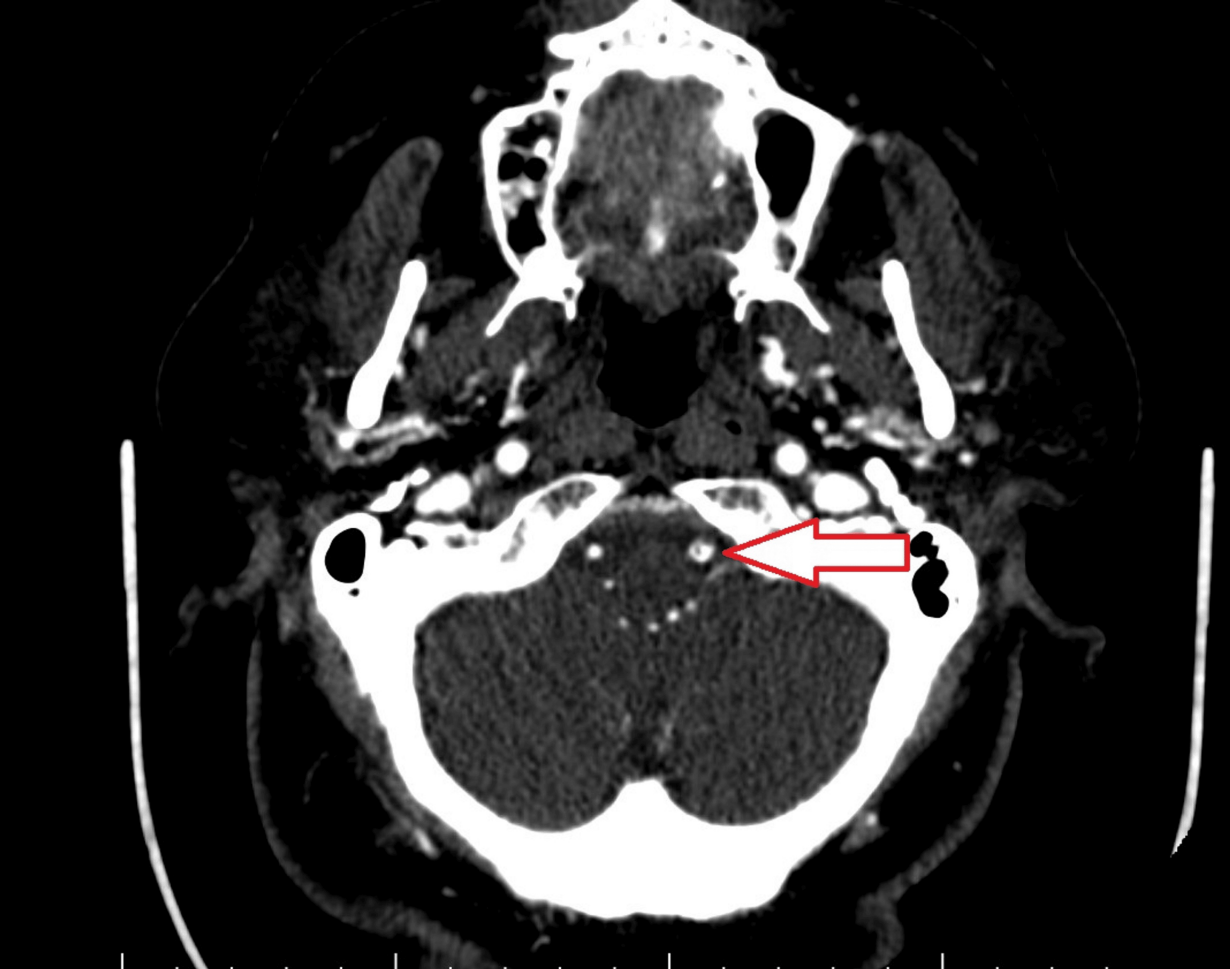
CT Angiography Showing Suspicious V4 Vertebral Artery Finding Computed tomography angiography (CTA) showing a thin hypodense flap in the V4 segment of the vertebral artery (arrow), raising suspicion for arterial dissection. Subsequent imaging did not confirm dissection.

Computed tomography angiography demonstrated a possible hypodense flap in the V4 segment of the vertebral artery, raising suspicion for dissection (Figure 3), though this was not confirmed. 

Given the atypical presentation, isolated cranial neuropathy with headache, and absence of radiological findings, CSF analysis was performed. The findings are summarized in Table 1, revealing mildly elevated opening pressure, lymphocytic pleocytosis, normal protein and glucose, and positive cryptococcal antigen. The relatively normal CSF profile with low cell count highlights the possibility of early or indolent cryptococcal infection.

**Table 1:** Cerebrospinal Fluid Analysis CRAG: cryptococcal antigen; cmH2O: centimeters of water

Parameter	Result	Reference Range
Opening pressure	26 cmH2O (mildly elevated)	10-20 cmH2O
Total cell count	7 cells/mm3	0-5 cells/mm3
Protein	33.1 mg/dl	15-45 mg/dl
Glucose	90 mg/dl	60-70% of serum glucose (serum glucose - 228 mg/dl)
CRAG	Positive	Negative

Additional investigations, including HIV testing and serum cryptococcal antigen, were negative. The summary of all investigations is presented in Table 2.

**Table 2:** Summary of Investigations MRA: magnetic resonance angiography; CTA: computed tomography angiography; CSF: cerebrospinal fluid; ANA-profile ( anti-U1 RNP/Sm): antinuclear antibody (ribonucleoprotein/Smith)-borderline positive (clinically insignificant)

Investigations	Findings
MRI Brain	Normal
MRA	No aneurysm
CTA	Suspected V4 flap
CSF	CRAG positive
HIV	Negative
ANA-RNP/SM	Borderline positive

Antinuclear antibody (ANA) profile was borderline positive and thus clinically insignificant. India ink staining and fungal culture were not performed due to resource limitations.

Rationale for diagnostic approach

Cerebrospinal fluid evaluation was undertaken due to atypical presentation: isolated cranial neuropathy, persistent headache, and absence of structural cause on imaging, and mildly elevated opening pressure. These findings warranted exclusion of infectious etiologies despite the absence of classical meningitis features.

Treatment

The patient was initiated on oral fluconazole therapy with therapeutic monitoring. While amphotericin B combined with flucytosine is the recommended induction therapy for cryptococcal meningitis, in view of the mild clinical presentation, absence of raised intracranial pressure-related complications, and resource considerations, the patient was managed with high-dose fluconazole following infectious disease consultation. Serial lumbar punctures were performed for intracranial pressure control. Glycemic status was optimized with insulin therapy. 

Outcome and follow-up

The patient demonstrated gradual improvement with resolution of ptosis and progressive recovery of ocular motility. At six weeks, there was near-complete resolution with minimal residual diplopia. The follow-up MRI was normal. Repeat CSF analysis demonstrated clearance of cryptococcal antigen. The cranial neuropathy resolved completely, and antifungal therapy was discontinued after clinical and laboratory resolution.

## Discussion

Cranial nerve involvement may result from basal meningeal inflammation, raised intracranial pressure, or direct fungal invasion. Cranial nerve palsies arise through multifactorial mechanisms, including inflammatory damage, ischemia, and mechanical compression along the nerve pathway. Cranial nerve involvement in cryptococcus is an established but under-recognized complication, often related to basal meningeal inflammation [2,4].

Cerebrospinal fluid cryptococcal antigen testing has high sensitivity and specificity (>95%) for the diagnosis of cryptococcal meningitis, although false positives are rare [4].

Cryptococcal meningitis typically presents with subacute neurological symptoms, but atypical presentations, including isolated cranial neuropathies, are increasingly reported [3-5]

Neurological complications of cryptococcal meningitis, including cranial neuropathies, are likely driven by a combination of host inflammatory response and persistent fungal burden [4,5].

Although sixth nerve palsy is more commonly reported, isolated oculomotor nerve involvement is rare but has been described in the literature [5]. Neuroimaging in this case (Figures 1-3) did not reveal a structural cause, prompting CSF analysis, which proved diagnostic (Table 1). 

The limitations in this case include the absence of India ink staining and fungal culture, and the lack of antigen titration. However, diagnostic confidence is supported by a compatible clinical presentation, elevated opening pressure, and therapeutic response with complete recovery.

Although diabetes is a strong risk factor for ischemic cranial neuropathy, the presence of headache, positive CSF cryptococcal antigen, and complete recovery with antifungal therapy argues against a microvascular etiology.

Learning points 

Pupil-sparing third nerve palsy should not be assumed to be purely ischemic, as infectious and inflammatory etiologies may present in a similar manner. Notably, cryptococcal meningitis can manifest as an isolated cranial neuropathy, even in the absence of classic systemic features. The presence of diabetes mellitus should not lead to premature diagnostic closure or anchoring bias, and clinicians must maintain a broad differential diagnosis. Therefore, cerebrospinal fluid analysis remains a crucial investigation in patients with atypical neuro-ophthalmic presentations to ensure accurate diagnosis and timely management.

## Conclusions

Pupil-sparing third nerve palsy does not reliably exclude serious underlying pathology. Cryptococcal meningitis can present as isolated cranial neuropathy without systemic features, even in patients without overt immunosuppression. Clinicians should maintain a high index of suspicion and a low threshold for cerebrospinal fluid analysis in atypical presentations to ensure timely diagnosis and appropriate management.

Clinical heuristics are valuable but must remain subordinate to evolving clinical evidence. The presence of diabetes mellitus in this patient exemplifies how cognitive biases, particularly diagnostic anchoring, can influence clinical reasoning and delay appropriate investigation. This case illustrates that the traditional dichotomy of pupil-involving versus pupil-sparing third nerve palsy is an oversimplification and should not replace comprehensive clinical evaluation. In the era of advanced neuroimaging, a normal scan should not preclude further evaluation when clinical features are atypical or evolving.

## References

[1] Geering K (1975). Lipase and unspecific esterase activity in the fat body of Aedes aegypti L. Acta Trop.

[2] Ahmadi S, Haghgoshayie E, Arjmand A, Hajebrahimi S, Hasanpoor E (2022). Patient safety improvement with the patient engagement in Iran: a best practice implementation project. PLoS One.

[3] Modi P, Singh J (2026 Jan-). Cranial nerve III palsy (oculomotor palsy). StatPearls [Internet].

[4] Pescador Ruschel MA, Thapa B (2026 Jan-). Cryptococcal meningitis. StatPearls [Internet].

[5] Nakahira S, Yamamoto M, Wood T (2025). An unusual clinical presentation of Cryptococcal meningitis: the importance of a detailed history and physical. IDCases.

